# Influence of physical activity home environment on fundamental movement skills development in Chinese preschoolers: mediating role of moderate-to-vigorous physical activity

**DOI:** 10.3389/fped.2024.1475263

**Published:** 2024-10-28

**Authors:** Pan Liu, Chengwen Fan, Fang Li, Zongyu Yang, Bin Yang, Long Yin

**Affiliations:** ^1^ Colleges of Physical Education, Hunan University of Technology, Zhuzhou, China; ^2^ School of Physical Education, Hunan First Normal University, Changsha, China; ^3^ School of Physical Education and Health Sciences, Guangxi University for Nationalities, Nanning, China; ^4^ College of Physical Education, Hunan Normal University, Changsha, China; ^5^ Research Base for Public Sports Services in Hunan Province, Changsha, China

**Keywords:** preschool children, fundamental movement skills, physical activity home environment, moderate-to-vigorous physical activity, locomotor skills, object control skills

## Abstract

**Background:**

Research on how the physical activity home environment affects fundamental movement skills (FMS) in preschool children in China is limited. However, the role of moderate-to-vigorous physical activity (MVPA) in this relationship is still unclear. This study aims to analyze gender differences in FMS, explore associations between the physical activity home environment, MVPA, and FMS, and investigate MVPA's mediating role in these relationships.

**Methods:**

We recruited 169 preschool children (95 boys, 74 girls; mean age 4.9 years) from four kindergartens in Hengyang, China. The Family Environment Scale on Motor Development for Preschool Urban Children (FESMPD) assessed physical activity home environment. Objective measurement of MVPA used ActiGraph wGT3-BT accelerometers. The Test of Gross Motor Development-3 (TGMD-3) evaluated FMS. Statistical analyses were conducted using the PROCESS macro in SPSS, with sociodemographic variables as controls.

**Results:**

Boys exhibited significantly higher levels of MVPA, parenting style, locomotor skills, and object control skills compared to girls (*P* < 0.05). MVPA, parenting style, and FMS showed positive correlations (*R* = 0.355–0.568, *P* < 0.05). Similarly, MVPA was positively correlated with the physical activity home environment (*β* = 0.237–0.568, *P* < 0.05). Parenting style emerged as a significant predictor of children's MVPA levels (*β* = 0.956, *P* < 0.001), and MVPA was a predictor of the development ofFMS and its subdomains (*β* = 0.097–0.207, *P* < 0.05). Furthermore, MVPA partially mediated the relationship between parenting style and the development of FMS in preschool children. The physical environment was also a significant predictor of children's MVPA (*β* = 0.637, *P* < 0.05), and in turn, MVPA predicted the development of FMS and its subdomains (*β* = 0.188–0.343, *P* < 0.01). Notably, MVPA fully mediated the relationship between the physical environment and the development of FMS.

**Conclusion:**

Overall, this study highlights the important roles of physical activity home environments and individual levels of MVPA in developing FMS in preschool children, noting significant gender differences. Parenting style greatly affects both MVPA and FMS development, while the physical environment fully mediates this relationship. Collaborative efforts among kindergartens, families, and communities are essential to support MVPA and improve FMS development.

## 1 Introduction

Fundamental movement skills (FMS) are essential for mastering advanced motor skills needed for sports and structured activities. FMS encompass two primary subcategories: locomotor skills (such as walking, running, jumping, and sliding) and object control skills (including throwing, catching, kicking, and dribbling) (1). However, contemporary public health challenges such as inadequate physical activity (PA), poor dietary habits, and prolonged sedentary behavior are increasingly prevalent among children and adolescents (2). These factors significantly hinder the development of FMS in this demographic (3), which plays a crucial role in combating childhood obesity and promoting overall health and well-being (4, 5). Rapid societal changes have reduced preschool children's physical activity time, delaying FMS acquisition (6). Given China's unique cultural context and large population, understanding the determinants influencing FMS in preschool children is imperative.

The family environment plays a crucial role in children's engagement in PA and the development of FMS (1), significantly influencing their behaviors and health outcomes (7). In this context, the Chinese government emphasized the significance of the family environment for children's holistic development in the “China's Children's Development Outline (2021–2030).” Michelle et al. (8) categorized the physical activity home environment into two dimensions: physical and social. The physical environment refers to the availability and accessibility of sports resources in the home. The social environment involves the roles and strategies parents use during physical activities, which are commonly referred to as parenting styles. Research shows a positive correlation between the family physical environment and FMS in preschoolers (9, 10). Interestingly, frequent acquisition of new toys does not necessarily correlate with improved FMS; instead, the type and quality of toys play a more critical role (10). Diverse toys are effective in engaging children and providing varied opportunities to practice motor skills, thereby promoting the development of different movement skills across the body (10). Children with stronger motor skills often demand more diverse toys and equipment (11). Studies also affirm that the quantity of toys and equipment at home correlates closely with the development of locomotor and object control skills in preschoolers (11).Additionally, adequate activity space encourages children to engage more willingly in physical activities. Compared to school physical education classes and playgrounds, the home environment offers a safer and more autonomous setting for physical expression (12). These findings underscore that a diverse array of toys and sufficient activity space at home significantly contribute to enhanced FMS development in preschool children. Furthermore, compared to other environments such as childcare centers and community settings, the physical activity home environment exerts a more significant influence on children's PA. Specifically, each additional type of play equipment is associated with a 5-minute increase in children's daily outdoor activity time (13, 14). This underscores that a supportive physical activity home environment not only enhances the development of children's FMS but also contributes to greater engagement in PA. Nevertheless, it remains unclear whether PA serves as a mediator in the relationship between the physical environment and the development of FMS.

Parenting styles play a crucial role in the development of FMS in children, particularly in preschoolers. Recent studies have increasingly focused on how parenting styles impact FMS in this age group. Morgan et al. highlighted that parents and immediate family members exert the most significant influence on enhancing FMS, given children spend a substantial portion of their leisure time at home outside of structured physical activities (15). Engaging in sports activities with children, encouraging exercise, and providing sports equipment and toys all positively influence FMS development in preschoolers (16–19). Greater parental support correlates with enhanced motor skills development, with research indicating varied effects on locomotor and object control skills among Chinese children (19). Positive parental attitudes towards physical activity are linked to improved locomotor skills but may affect object control skills differently (19). Moreover, studies demonstrate that parents significantly boost children's participation in MVPA through encouragement, direct involvement, and supervision, indirectly supporting the development of FMS (15, 20–22).

MVPA is widely recognized for its close association with the development of FMS in preschool children (20, 22–24). The conceptual model proposed by Stodden et al. (25) suggests a reciprocal and dynamic relationship between PA and FMS, where PA influences the early development of FMS, and mastery of FMS supports sustained engagement in PA through adulthood. Longitudinal research by Barnett et al. (22) has shown that MVPA at age 3.5 positively predicts locomotor skills at age 5, though it does not predict object control skills at any age. There is ample evidence that the physical activity home environment influences FMS development in preschoolers. MVPA also plays an important role in shaping FMS and its subcategories. However, it is still unclear whether parenting styles impact FMS through MVPA (19, 24, 26–28). Notably, there is currently a lack of research investigating the relationship between parenting styles and FMS among Chinese preschool children, especially regarding the potential mediating role of MVPA (19, 24, 26–28). Furthermore, influenced by traditional beliefs, many Chinese parents adopt a controlling parenting style that may prioritize academic achievements over physical development and lack empathy towards children. Therefore, it is crucial to understand the potential role of MVPA in the relationship between parenting style and the development of FMS in Chinese children.

Research indicates that Chinese children demonstrate significantly lower levels of gross motor skills (e.g., catching and throwing) compared to their counterparts in the UK and the US (29, 30). Insufficient development of FMS during early childhood not only negatively impacts physical health, mental well-being, and social adaptation (31), but also diminishes the likelihood of participating in physical activity in adolescence and adulthood, thereby elevating the risk of overweight and obesity (32). Furthermore, societal expectations related to gender often result in parents inadvertently providing more care and physical activity opportunities to boys. This suggests that there may be gender-specific variations in FMS development. Identifying the factors that influence FMS development and understanding gender differences are crucial for advancing FMS in Chinese preschool children.

**Figure 1:**
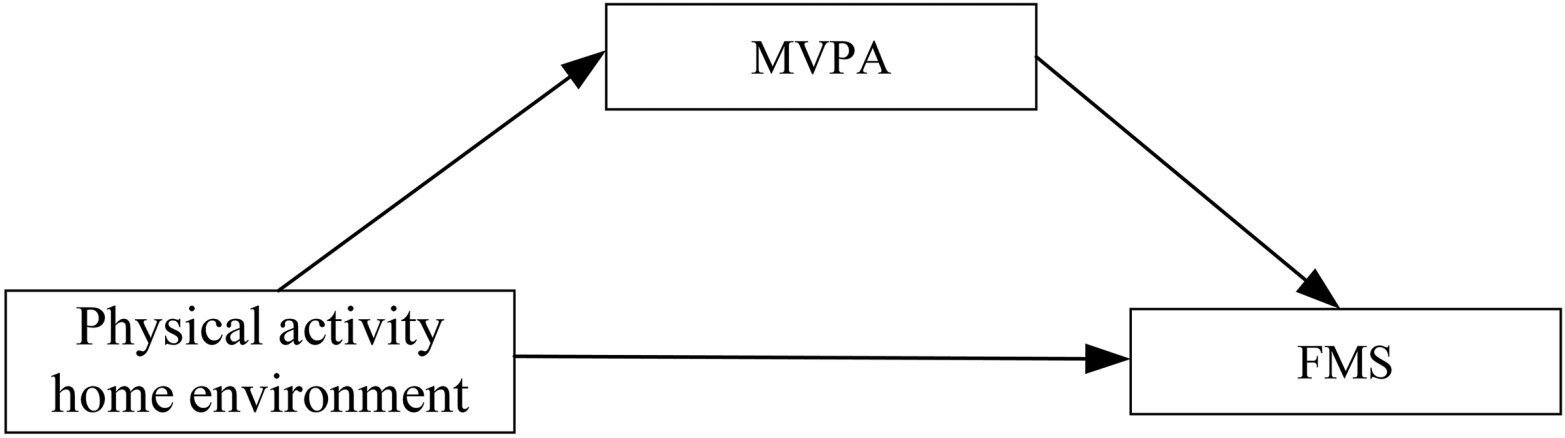
Hypothetical model of moderate-to vigorous-intensity physical activity as a mediator of the relationship MVPA, moderate-to-vigorous physical activity; FMS, fundamental movement skills.

While previous research has demonstrated the influence of the physical activity home environment and MVPA on the development of FMS in preschool children, it remains uncertain whether MVPA mediates the relationship between the physical activity home environment and FMS development. This question is particularly relevant given the unique cultural context and educational philosophies in China. This study aims to examine the relationships among the physical activity home environment, MVPA, and FMS in Chinese preschool children. The study aims to investigate the following: (1) the gender differences in FMS among preschool children; (2) the associations between the physical activity home environment, MVPA and FMS; and (3) the mediating role of MVPA in the relationship between physical activity home environment and FMS (see Figure 1). Answering these questions will offer insights to guide parental practices in fostering motor skills and promoting children's health. Furthermore, the findings will offer scientific evidence to inform the development of targeted interventions by governments and educational institutions. Thereby enhancing the collaborative efforts of families, schools, and society in supporting the comprehensive development of children.

## 2 Methods

### 2.1 Design and participants

**Figure 2:**
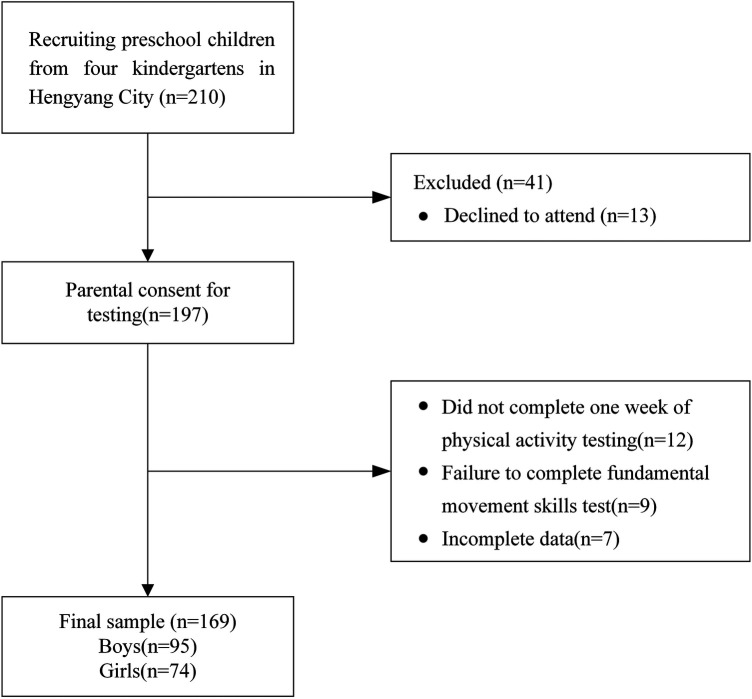
Process of recruitment and participant selection.

This study uses a cross-sectional design and employs convenience sampling based on geographical location, socioeconomic status, and physical education methods. Researchers selected 4 kindergartens from the main urban area of Hengyang City, Hunan Province, China. Each kindergarten is accredited by the Hengyang City Education Bureau as an early childhood education and care (ECEC) institution. To ensure a representative sample across various educational sectors, the study includes kindergartens from 4 distinct educational districts within the main urban area, with 1 kindergarten randomly chosen from each district, yielding a total of 4 kindergartens. Each institution included classes for nursery class, middle class and senior class, with a total of 210 registered preschool children invited to participate in the study. Prior to formal testing, researchers distributed informed consent forms to parents, briefly outlining the study's purpose and procedures. The aim was to obtain parental permission for their children's participation in the study, resulting in consent from 197 parents. During the physical activity test, 12 children wore accelerometers for less than a week, and the valid data collected was less than 3 days, leading to missing data. Furthermore, 9 children did not complete the entire testing protocol for fundamental motor skills, including all tasks for locomotor and object control skills. As a result, 7 sets of invalid data were excluded during the data processing stage. Ultimately, 169 valid samples were retained, comprising 95 boys and 74 girls, with an average age of 4.9 years (SD = 0.75) (see Figure 2). This research protocol was approved by the Ethics Committee of Hengyang Normal University in 2021 (NO.2021003).

### 2.2 Measures

#### 2.2.1 Physical activity home environment

The validated Family Environment Scale on Motor Development for Preschool Urban Children (FESMDP) was utilized as the primary survey tool in this study (33). To meet the study's objectives, the scale was adjusted to focus on two dimensions: home physical environment and parenting style. The home physical environment was assessed across outdoor space (6 items), indoor space (6 items), fine motor toys (8 items), and gross motor toys (8 items). Each item included pictures for parents to accurately recognize and categorize, with scores assigned as 1 (presence of the equipment) or 0 (absence of the equipment). The scores of the 4 items were summed to obtain the physical environment score, with higher scores indicating a better physical environment. Parenting style was evaluated using 18 items that described parents’ behaviors and attitudes towards daily self-care and activities involving children. Responses were rated on a 5-point scale ranging from 1 (never) to 5 (always). The cumulative score of these items reflected the overall score for parenting behavior, with higher scores indicating more favorable parenting styles.

The survey was administered electronically within the kindergarten setting, where parents completed and submitted the questionnaire online. Collected data were subsequently downloaded by the research team, underwent thorough cleaning, and were scored according to predefined criteria to ensure the accuracy and reliability of the findings.

#### 2.2.2 MVPA

MVPA was objectively assessed using the Actigraph wGT3X-BT accelerometer, and data extraction was performed using ActiLife software (Version 6.13.3 from Actigraph Corps, Pensacola, Florida, USA) with a sampling interval of 15 s (34). Prior to formal testing, a subset of children underwent a one-week pre-test to optimize the placement and timing of accelerometer wear, ensuring accurate and reliable data collection. Based on pre-test results, accelerometers were worn on the right hip for seven consecutive days, excluding swimming, sleeping, and bathing to prevent damage. Wear time algorithms, as recommended by Choi et al. (35), were applied, requiring a minimum of 8 h of wear per day and at least 3 valid days (including 2 weekdays and 1 weekend day) for data to be considered valid for the study. Consistent with guidelines by Butte et al. (34), PA levels were categorized using ActiLife software into low-intensity physical activity (LPA, 240–2119 counts/epoch), moderate-intensity physical activity (MPA, 2,120–4,449 counts/epoch), and vigorous-intensity physical activity (VPA, ≥4,450 counts/epoch), where MVPA was calculated as the sum of MPA and VPA. Childcare staff and parents received detailed verbal and written instructions on accelerometer wear, removal procedures, and were encouraged to maintain daily wear logs to record wear times and conditions.

#### 2.2.3 FMS

FMS were assessed using the Test of Gross Motor Development-3 (TGMD-3), a tool widely utilized in China and suitable for the current study population (36). FMS were categorized into two subdomains: locomotor skills and object control skills. For clarity, this study refers to fundamental movement skills as total motor skills (Total MS). The locomotor skills assessment comprised six subtests, while the object control skills assessment included seven subtests. The testing procedures strictly adhered to the TGMD-3 standards, with trained assessors demonstrating each task followed by participants practicing to ensure readiness before formal testing commenced. All sessions were recorded, and these videos served as the basis for subsequent scoring.

After testing, trained researchers evaluated performance according to TGMD-3 criteria. Each subtest had 3–5 scoring criteria, with scores of 1 awarded for meeting criteria and 0 for not meeting them. Any scoring disputes were resolved through consensus among researchers to determine final scores. Scores from each subtest were summed to yield totals for locomotor and object control skills. The maximum score for locomotor skills was 46, for object control skills was 54, and the Total MS score represented the sum of locomotor and object control skills scores, with a maximum score of 100 indicating better overall FMS performance in children.

#### 2.2.4 Demographic characteristics

Upon obtaining parental consent, demographic information was gathered using a questionnaire completed by parents, which included the child's age and gender. Additionally, kindergarten health personnel recorded the child's height and weight, from which body mass index (BMI) was calculated using the formula weight/height² (kg/m²) to assess body weight status. Family socioeconomic status (SES), a recognized factor influencing children's FMS based on prior studies (9, 17), was assessed through parental reports of educational attainment, occupations, and monthly household income.

SES was determined using principal component analysis and categorized into three levels (low, medium, high), which were incorporated as covariates in the subsequent data analysis process.

#### 2.2.5 Statistical analysis

In the preliminary data analysis phase, outliers and extreme values were addressed using interpolation techniques. Data were analyzed with SPSS 27.0 (IBM Corp., Armonk, NY, USA). Initially, descriptive statistics (M ± SD) were computed for the physical activity home environment, moderate-to-vigorous physical activity (MVPA), and fundamental movement skills (FMS) of preschoolers. Independent samples *t*-tests were then conducted to examine gender differences in these variables.

Before examining mediation effects, the study first investigates the relationships among the physical activity home environment, MVPA and FMS in preschool children through correlational analysis. This step identifies variables suitable for further analysis. To explore the mediating role of MVPA between parenting style and FMS, the study controls for age, gender, SES and BMI as covariates. The mediation effects are assessed using the PROCESS macro in SPSS27.The study employs the bias-corrected non-parametric percentile bootstrap method, performing 5,000 resamples to compute the 95% confidence interval for the mediation effect. Bootstrap methods are extensively used and yield reliable results for testing mediation effects (37, 38). A significant mediation effect is indicated when the lower limit (LLCI) and upper limit (ULCI) of the confidence interval do not include 0 (38, 39). A simple mediation model (Model 4) is applied to test the mediating effect of MVPA between PS and FMS. All statistical analyses are conducted with a significance level set at *P* < 0.05.

## 3 Results

### 3.1 Descriptive statistics of physical activity home environment, MVPA, and FMS in preschool children

The independent sample *t*-test indicated significant gender differences in MVPA (*t* = 3.983, *P* < 0.001), parenting style (*t* = 3.239, *P* < 0.01), locomotor skills (*t* = 2.424, *P* < 0.05), object control skills (*t* = 4.763, *P* < 0.001), and total motor skill (*t* = 3.979, *P* < 0.001). As shown in Table 1, boys had significantly higher levels of moderate-to-vigorous physical activity, parenting style, locomotor skills, object control skills, and total motor skills compared to girls.

**Table 1:** Descriptive statistics of physical activity home environment, physical activity, and fundamental movement skills (*n* = 169).

Variables	Total	Boys	Girls
	*n* = 169 M(SD)	*n* = 95 M(SD)	*n* = 74 M(SD)
Age	4.9 (0.8)	5.0 (0.8)	4.9 (0.7)
BMI	15.7 (1.6)	15.7 (1.6)	15.6 (1.5)
MVPA	49.7 (14.6)	53.5 (15.6)***	44.9 (11.6)
Parenting Style	72.8 (7.9)	74.5 (8.1)**	70.6 (7.1)
Physical Environment	20.1 (3.9)	20.6 (3.9)	19.4 (3.9)
Locomotor Skills	29.1 (7.0)	30.3 (7.5)*	27.7 (6.2)
Object Control Skills	27.9 (7.1)	30.0 (7.7)***	25.1 (5.1)
Total Motor Skill	57.0 (13.0)	60.3 (13.1)***	52.8 (10.9)

Note: *, ** and *** indicate significant differences between boys and girls on this indicator, *P* < 0.05, *P* < 0.01 and *P* < 0.001, respectively. M, mean; SD, standard deviation.

### 3.2 Correlation analysis between physical activity home environment, MVPA, and FMS in preschool children

As shown in Table 2, MVPA in preschool children exhibited significant moderate positive correlations with locomotor skills, object control skills, total motor skills, and parenting style (*R* = 0.447, 0.444, 0.494, 0.568), and a significant low positive correlation with the physical environment (*R* = 0.237). Locomotor skills were significantly and moderately correlated with parenting style (*R* = 0.460), while object control skills also showed a significant moderate correlation with parenting style (*R* = 0.355). Additionally, total motor skills demonstrated a significant moderate positive correlation with parenting style (*R* = 0.451).

**Table 2:** Correlation analysis of physical activity home environment, physical activity, and fundamental movement skills.

	1	2	3	4	5
1. MVPA	1				
2. Locomotor skills	0.447**	1			
3. Object control skills	0.444**	0.627**	1		
4.Total motor skill	0.494**	0.901**	0.903**	1	
5. Parenting style	0.568**	0.460**	0.355**	0.451**	1
6. Physical environment	0.237**	0.080	0.138	0.121	0.304**

Note: * and ** indicate significant correlation at *P* < 0.05 and *P* < 0.01, respectively.

### 3.3 Effects of physical activity home environment and MVPA on FMS in preschool children

**Figure 3:**
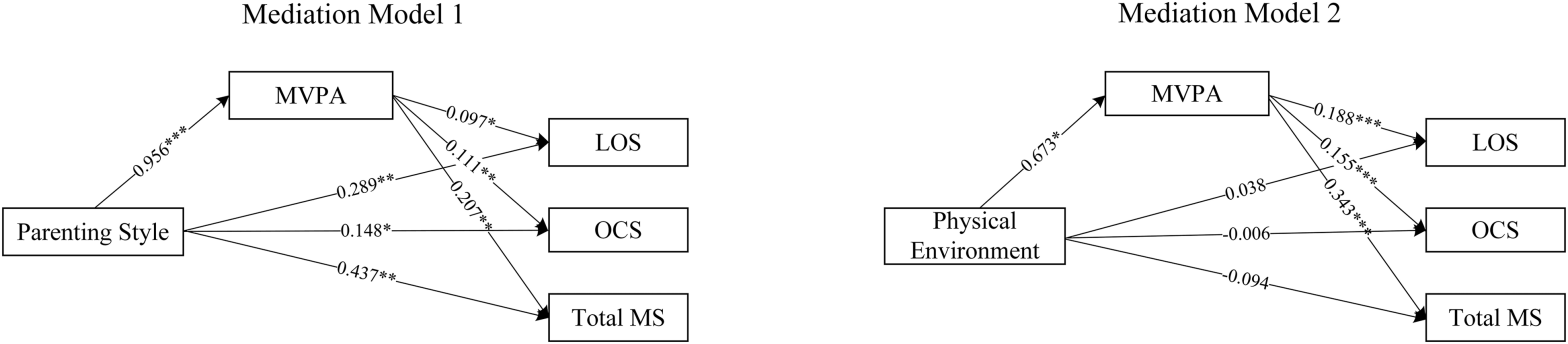
The mediation effect of Moderate-to-Vigorous physical activity between parenting styles and fundamental movement skills. MVPA, moderate-to-vigorous physical activity; LOS, locomotor skills; OCS, object control skills; Total MS, total motor skills.

Figure 3 shows that parenting style predicted MVPA (*β* = 0.956, *P* < 0.001), which significantly predicted locomotor skills (*β* = 0.097, *P* < 0.05), object control skills (*β* = 0.111, *P* < 0.01), and total motor skills (*β* = 0.207, *P* < 0.01). Parenting style directly predicted locomotor (*β* = 0.289, *P* < 0.05), object control skills (*β* = 0.148, *P* < 0.05), and total motor skills (*β* = 0.437, *P* < 0.01). The physical environment positively influenced MVPA (*β* = 0.673, *P* < 0.05), which significantly predicted locomotor (*β* = 0.188, *P* < 0.001), object control skills (*β* = 0.155, *P* < 0.001), and total motor skills (*β* = 0.343, *P* < 0.001). However, the physical environment did not have a direct significant effect on any of the motor skills.

Table 3 shows parenting style had a total effect of 0.382 on locomotor skills, with a direct effect of 0.289 [95% CI (0.156, 0.423)]. The indirect effect through MVPA was 0.092 [95% CI (0.015, 0.184)], indicating that MVPA partially mediated this relationship. For object control skills, the total effect of parenting style was 0.254, with a direct effect of 0.148 [95% CI (0.017, 0.279)] and an indirect effect of 0.118 [95% CI (0.039, 0.205)], showing that MVPA also partially mediated this relationship. Regarding total motor skills, the total effect of parenting style was 0.625, with a direct effect of 0.437 [95% CI (0.213, 0.661)] and an indirect effect of 0.198 [95% CI (0.076, 0.349)], indicating that MVPA also partially mediated this relationship.

**Table 3:** Mediation effect of moderate-to-vigorous physical activity between physical activity home environment and fundamental movement skills.

	Type of effect	Path	Effect value (standard error)	95% CI	Effect size
Model 1	Total effect		0.382*** (0.582)	[0.267–0.497]	
	Direct effect	PS→LOS	0.289*** (0.068)	[0.156–0.423]	75.65%
	Indirect effect	PS→MVPA→LOS	0.092** (0.043)	[0.015–0.184]	24.08%
	Total effect		0.254*** (0.057)	[0.140–0.367]	
	Direct effect	PS→OCS	0.148* (0.066)	[0.017–0.279]	58.27%
	Indirect effect	PS→MVPA→OCS	0.118** (0.041)	[0.039–0.205]	46.46%
	Total effect		0.625*** (0.099)	[0.440–0.830]	
	Direct effect	PS→Total MS	0.437** (0.113)	[0.213–0.661]	69.92%
	Indirect effect	PS→MVPA→Total MS	0.198** (0.069)	[0.076–0.349]	31.68%
Model 2	Total effect		0.038 (0.128)	[−0.215–0.291]	
	Direct effect	PE→LOS	−0.088 (0.120)	[−0.326–0.149]	
	Indirect effect	PE→MVPA→LOS	0.126** (0.051)	[0.037-–0.235]	
	Total effect		0.098 (0.118)	[−0.135–0.332]	
	Direct effect	PE→OCS	−0.006 (0.113)	[−0.230–0.218]	
	Indirect effect	PE→MVPA→OCS	0.104** (0.043)	[0.029–0.197]	
	Total effect		0.137 (0.216)	[−0.290–0.562]	
	Direct effect	PE→Total MS	−0.094 (0.199)	[−0.488–0.299]	
	Indirect effect	PE→MVPA→Total MS	0.231** (0.090)	[0.067–0.416]	

Note: Bootstrap sampling was conducted 5,000 times with a 95% confidence interval. *** indicates *P* < 0.001, ** indicates *P* < 0.01, and * indicates *P* < 0.05. (LOS, locomotor skills; OCS, object control skills; Total MS, total motor skill; PS, parenting style; PE, physical environment; MVPA, moderate-to-vigorous physical activity).

In the relationship between the physical environment and FMS, MVPA served as a full mediator. The total effect of the physical environment on locomotor skills was 0.038, with a non-significant direct effect of −0.088 [95% CI (−0.326, 0.149)] and a significant indirect effect through MVPA of 0.126 [95% CI (0.037, 0.235)]. For object control skills, the total effect of the physical environment was 0.098, with a non-significant direct effect of −0.006 [95% CI (−0.230, 0.218)] and a significant indirect effect of 0.104 [95% CI (0.029, 0.197)], indicating that MVPA fully mediated this relationship. Lastly, the total effect of the physical environment on total motor skills was 0.137, with a non-significant direct effect of −0.094 [95% CI (−0.488, 0.299)] and a significant indirect effect of 0.231 [95% CI (0.067, 0.416)], showing that MVPA also fully mediated this relationship.

## 4 Discussion

This study aimed to examine the relationships between the physical activity home environment, MVPA, and FMS in Chinese preschool children, while also exploring the mediating role of MVPA between the physical activity home environment and FMS. The findings revealed significant gender differences in FMS. Additionally, there were notable positive correlations between MVPA and locomotor skills, object control skills, total motor skills, parenting style and the physical environment. FMS showed significant positive correlations with parenting style. Parenting style had a significant positive effect on locomotor skills, object control skills, and total motor skills, with MVPA partially mediating the relationship between parenting style and FMS. Furthermore, the physical environment significantly influenced MVPA, with MVPA fully mediating the relationship between the physical environment and FMS.

### 4.1 Gender differences in FMS

The study identifies significant gender differences in FMS, encompassing both locomotor and object control skills. Specifically, boys demonstrate higher scores in FMS compared to girls, which aligns with previous research (40, 41). These observed differences may be attributed to various social and environmental factors as well as divergent preferences for physical activities (42). Firstly, the types of physical activities engaged in by preschool children are shaped by multiple influences, including family dynamics, educator interactions, peer relationships, and the physical environment (43). Societal expectations concerning gender roles often result in boys receiving more encouragement and opportunities for physical success, potentially leading to a greater emphasis on physical exercise for boys (40, 44). In contrast, girls are more inclined towards rhythmic activities such as dance, while boys prefer team sports involving physical contact, such as ball games (45). Additionally, boys generally exhibit more advanced physical development, including increased size and strength, which may further facilitate their proficiency in object control skills (46). Notably, when assessing overall motor skills (encompassing both locomotor and object control skills) boys consistently outperform girls. This finding highlights the necessity of targeted interventions aimed at improving object control skills among girls. Therefore, future strategies for enhancing children's FMS should carefully consider gender-specific differences to devise more effective and tailored intervention programs.

### 4.2 Relationship among physical activity home environment and MVPA

This study categorizes the home physical environment into two dimensions: physical environment and parenting style. It found that physical environment and parenting styles are correlated with MVPA in preschool children, aligning with findings from Tandon et al. (20). Tandon et al. noted that having sports equipment at home, such as basketball hoops, can increase children's MVPA levels (20). In this study, physical environment encompass toys that enable climbing, crawling, rolling, building, and controlling ride-on vehicles (33). While there are differences from Tandon's research, the relevance persists: Tandon's study focused on children aged 6–11 years, whereas this study concentrated on preschool children under 6 years old. Unlike specialized sports equipment, a diverse array of toys can engage preschoolers’ attention and interest (47).

Moreover, the study identified that parents’ own PA levels, support for children's PA participation, and joint PA with children influence children's PA levels (48), consistent with this study's finding of a significant correlation between parenting style and children's MVPA. Additionally, research indicates that fathers are more likely to increase their daughters’ PA time (15), as they tend to actively engage children in varied PA types marked by lively, stimulating, and adventurous interactive play (49, 50). This underscores that fostering a positive physical activity home environment effectively enhances children's PA levels.

### 4.3 Relationship among physical activity home environment and FMS

The development of FMS in preschool children is pivotal for their physical and mental well-being (4, 51). This study explores the relationship between FMS, categorized into locomotor skills and object control skills, and the physical activity home environment. It reveals significant correlations between preschool children's FMS and its subdomain with parenting styles, consistent with prior research (10, 19). According to the social ecological model, parenting styles exert a decisive influence on children's behavior (52). The acquisition of children's FMS is not merely innate but often relies on parental guidance and role modeling, which are crucial for skill development (53). Insufficient parental emphasis on motor development can hinder skill acquisition.

Furthermore, the study identifies correlations between physical environment and the development of preschool children's FMS. This aligns with findings from Cools et al. (10), who observed that a variety of sports equipment at home contributes to preschool children's FMS development. A randomized controlled trial additionally indicated that interventions enhancing interactive playtime between parents and children, coupled with access to portable gaming facilities at home, significantly enhance preschool children's FMS (54). Notably, this study also establishes a link between fine motor toys and preschool children's object control skills. Consistent with other research, studies indicate that toys such as building blocks can improve children's small muscle groups and eye coordination, thereby supporting fine motor skill development (55). Fine motor skills are crucial for laying the groundwork for object control skills (56), highlighting their importance in skill development. Collectively, these findings underscore that, alongside increasing physical activity levels, a diverse array of play opportunities can foster the development of FMS in preschool children.

### 4.4 Mediating role of MVPA between physical activity home environment and FMS

This study found that MVPA partially mediates the relationship between parenting style and preschool children's FMS, while fully mediating the relationship between the physical environment and FMS. These findings suggest that the physical activity home environment can promote the development of FMS by increasing MVPA levels in preschool children.

The socio-ecological model posits that the development of FMS is influenced by various factors and contextual characteristics, including parental attributes and support. Prior research has established that parental support and their own physical activity levels positively correlate with children's motor skill development and MVPA (10, 16, 57). When parents display positive attitudes and an interest in physical activity, their children are more likely to develop confidence and self-efficacy (52). This positive interaction enhances the physical activity levels of both parents and children, which, in turn, supports motor skill development (40). Numerous studies affirm that physical activity, particularly MVPA, is crucial for FMS development (22–24). Increased engagement in physical activity enhances neuromotor development, which further promotes FMS (25), creating a dynamic, reinforcing model. Furthermore, children involved in physical activities often receive guidance from peers or teachers, facilitating the acquisition of motor skills (58). Goodway et al. (1) highlighted that FMS development in preschool children requires structured teaching, practice, and reinforcement rather than occurring naturally with age. However, in China, traditional educational values often prioritize cognitive development over physical activity, leading to an increasingly academic-focused educational model. This sedentary approach places excessive academic pressure on children, reducing physical activity levels and slowing motor skill development. Research also suggests that fathers’ involvement in physical activity with their children is more effective in promoting object control skills compared to mothers (59), though the impact on locomotor skills remains unclear. Although this study confirms the role of MVPA in both locomotor and object control skills, it does not address the differential effects of physical activity by fathers vs. mothers on children's FMS. Future research should explore and compare the impacts of fathers’ and mothers’ physical activities on children's FMS.

Furthermore, this study revealed that MVPA fully mediates the relationship between the physical environment and FMS in preschool children. To our knowledge, this finding has not been previously reported. This suggests that improvements in children's FMS are primarily driven by increases in PA levels, rather than the direct influence of the physical environment. Although previous studies have confirmed that the physical environment can directly affect FMS development, its role appears secondary (9, 10). Research has demonstrated that a diverse range of toys and equipment can stimulate higher PA levels in children (13, 14), and increased PA provides more opportunities for engagement in gross motor activities, thereby enhancing FMS development (21, 22). These findings highlight that optimizing the physical environment to promote higher MVPA levels is an effective strategy for improving children's FMS. In summary, this study confirms the indirect role of MVPA in the development of FMS in preschool children. Looking ahead, fostering a supportive physical activity home environment, while emphasizing increased MVPA participation, will be crucial for promoting the comprehensive development of preschool children.

### 4.5 Study strengths and limitations

This study represents the first exploration into the relationship among physical activity home environment, MVPA and FMS in Chinese preschool children, while also verifying the mediating role of MVPA between parenting styles and FMS. The findings provide theoretical insights that can guide policymakers in refining intervention measures. The study's strengths include the objective measurement of PA in preschool children, which overcomes biases associated with subjective questionnaires. Additionally, the use of the TGMD-3 administered by trained professionals to assess FMS enhances the study's credibility. However, there are several limitations to consider. Firstly, the physical activity home environment was assessed using parent-completed questionnaires, potentially introducing recall bias. Secondly, the cross-sectional nature of the study limits its ability to establish causal relationships between variables. Future research should adopt longitudinal designs to further elucidate these causal relationships. Additionally, this study was conducted in a large city in the central-southern region of China, and the impact of COVID-19 further reduced the final effective sample size. Consequently, the findings may not be generalizable beyond this specific context.

## 5 Conclusion

This study identified significant gender differences in FMS development among preschool children, with strong positive associations between the physical activity home environment, MVPA, and FMS. Parenting style positively influenced both MVPA and FMS, while the physical environment impacted MVPA. MVPA partially mediated the relationship between parenting style and FMS, and fully mediated the relationship between the physical environment and FMS. These findings highlight the need to consider school environments, gender differences and the roles of physical activity home environment and MVPA. Additionally, policies should be culturally tailored to promote active lifestyles, addressing local values and beliefs. Collaborative efforts from preschools, families, and children are essential for effective FMS development.

## Data Availability

The raw data supporting the conclusions of this article will be made available by the authors, without undue reservation.
